# Electrophysiological Rotor Ablation in *In-Silico* Modeling of Atrial Fibrillation: Comparisons with Dominant Frequency, Shannon Entropy, and Phase Singularity

**DOI:** 10.1371/journal.pone.0149695

**Published:** 2016-02-24

**Authors:** Minki Hwang, Jun-Seop Song, Young-Seon Lee, Changyong Li, Eun Bo Shim, Hui-Nam Pak

**Affiliations:** 1 Division of Cardiology, Yonsei University Health System, Seoul, Republic of Korea; 2 Department of Mechanical and Biomedical Engineering, Kangwon National University, Chuncheon, Kangwon-do, Republic of Korea; Gent University, BELGIUM

## Abstract

**Background:**

Although rotors have been considered among the drivers of atrial fibrillation (AF), the rotor definition is inconsistent. We evaluated the nature of rotors in 2D and 3D *in- silico* models of persistent AF (PeAF) by analyzing phase singularity (PS), dominant frequency (DF), Shannon entropy (ShEn), and complex fractionated atrial electrogram cycle length (CFAE-CL) and their ablation.

**Methods:**

Mother rotor was spatiotemporally defined as stationary reentries with a meandering tip remaining within half the wavelength and lasting longer than 5 s. We generated 2D- and 3D-maps of the PS, DF, ShEn, and CFAE-CL during AF. The spatial correlations and ablation outcomes targeting each parameter were analyzed.

**Results:**

1. In the 2D PeAF model, we observed a mother rotor that matched relatively well with DF (>9 Hz, 71.0%, p<0.001), ShEn (upper 2.5%, 33.2%, p<0.001), and CFAE-CL (lower 2.5%, 23.7%, p<0.001). 2. The 3D-PeAF model also showed mother rotors that had spatial correlations with DF (>5.5 Hz, 39.7%, p<0.001), ShEn (upper 8.5%, 15.1%, p <0.001), and CFAE (lower 8.5%, 8.0%, p = 0.002). 3. In both the 2D and 3D models, virtual ablation targeting the upper 5% of the DF terminated AF within 20 s, but not the ablations based on long-lasting PS, high ShEn area, or lower CFAE-CL area.

**Conclusion:**

Mother rotors were observed in both 2D and 3D human AF models. Rotor locations were well represented by DF, and their virtual ablation altered wave dynamics and terminated AF.

## Introduction

Atrial fibrillation (AF) is a cardiac electrophysiological rhythm disorder that results in the absence of normal atrial contractions. Although catheter ablation of atrial fibrillation is an effective rhythm control strategy, the recurrence rate is still significant [1]. The mechanism of cardiac fibrillation is still poorly understood, and two major theories exist: the focal source hypothesis [2] and the multiple wavelet hypothesis [3–6]. While the focal source hypothesis explains cardiac fibrillation in terms of stable periodic sources (such as mother rotors) that induce wave breakup, the multiple wavelet hypothesis is predicated on the existence of multiple wavelets that may shrink, collide with other wavelets, or create new rotors [7].

A rotor is a vortex of electrical waves that is either stationary or drifting. The term “mother rotor” is used when wavelets are generated from the breakup of the wavefronts of the rotor [7, 8]. The mother rotor, which is stable and induces fibrillatory conduction, is considered a mechanism responsible for the maintenance of AF [8–11]. An example of a temporal definition of mother rotor is the one used by Keldermann et al. [12], which defines the duration of a ventricular fibrillation (VF) mother rotor as at least 5 s. Both temporal and spatial definitions of the mother rotor would help quantify the results of studies investigating the mechanism of cardiac fibrillation.

In a previous study [13], we examined the relationships among fibrillation wave dynamics parameters using a ventricular model. In this study, we focused on rotors and attempted to identify the wave dynamics parameter that best represent rotors in atrial fibrillation using an atrial model. We defined the mother rotor both spatially and temporally based on concepts described in the literature [7, 8, 12]. We also examined ablation methods using computer simulation in an attempt to identify ablation approaches that can eliminate the mother rotor. We performed virtual ablation targeting the mother rotor using both 2D and 3D models based on multiple analytical parameters such as phase singularities (PS), dominant frequency (DF), Shannon entropy (ShEn), and complex fractionated atrial electrogram cycle length (CFAE-CL) and compared the performance of each ablation method. We chose these parameters because they are frequently used by many researchers to trace rotors and examine the wave dynamics [14–16].

## Methods

### Two-Dimensional AF Modeling

For the 2D simulation of atrial cardiac wave propagation, the following reaction-diffusion equation was solved using finite element method:

$$\frac{\partial V_{m}}{\partial t}=\frac{1}{\beta C_{m}}\{\nabla \cdot D\nabla V_{m}-\beta (I_{ion}+I_{s})\}$$ (1)

where *Vm* is the membrane potential, β is the membrane surface-to-volume ratio, C_m_ is the membrane capacitance per unit area, D is conductivity tensor, and I_ion_ and I_s_ are, respectively, the ion current and stimulation current. The computational domain consisted of 600 × 600 elements. The size of each element was 250 μm × 250 μm. The ionic currents in each cell were determined using the human atrial action potential (AP) model developed by Courtemanche et al. [17]. Reentry was initiated by setting the membrane potential to 0 mV on half of the sheet perpendicular to the linear wave generated by a line stimulation at the side of the sheet. Ionic currents were adjusted to represent control and persistent AF (PeAF) conditions as shown in Table 1. These ionic currents were modified from a previous report of PeAF conditions [18] to allow AF to be sustained for longer than 30 s. We searched and found the conditions under which AF is sustained for longer than 30 s.

**Table 1:** Maximal ion current conductance or maximal ion current for mother rotor induction.

		Control	AF
Maximal Conductance (nS/nF)			
	I_Na_	7.8	7.8*0.9
	I_to_	0.1652	0.1652*0.3
	I_CaL_	0.1238	0.1238*0.5
	I_K1_	0.09	0.09*2
	I_Kur_	*g_Kur_	*g_Kur_*0.5
Maximal Current (pA/pF)			
	I_NaCa(max)_	1,600	1,600*1.4
	I_up(max)_	0.005	0.005*1.25

*g_Kur_ = $0.005+\frac{0.05}{1+exp\left[\frac{\left(V-15\right)}{-13}\right]}$, where V is membrane potential (mV).

### Determination of PS, DF, ShEn, and CFAE

The PS, DF, and CFAE were determined as described previously by Yun et al. [13]. Briefly, PS was defined as the point at which the phase of the AP is undetermined, and was detected using Iyer-Gray’s algorithm [19]. To determine the DF, the power spectral density was obtained by Fourier transform of each cell’s virtual AP, and the DF was defined as the frequency at which the power was highest. To generate CFAE, we used a virtual bipolar catheter consisting of two rectangular electrodes located 1.0 mm apart simulating a commercially available ablation catheter (Thermocool, Johnson & Johnson Inc., Diamond Bar, CA, USA) [13]. The sizes of the distal and proximal electrodes were 3.5 × 2.0 mm and 1.5 × 2.0 mm, respectively. The CFAE-CL was obtained by averaging the time intervals between consecutive local minimum of time derivatives of virtual bipolar electrograms over a duration of 6 s. ShEn was calculated as described previously by Ganesan et al. [15].

### Quantitative Definition of Mother Rotor

**Fig 1:**
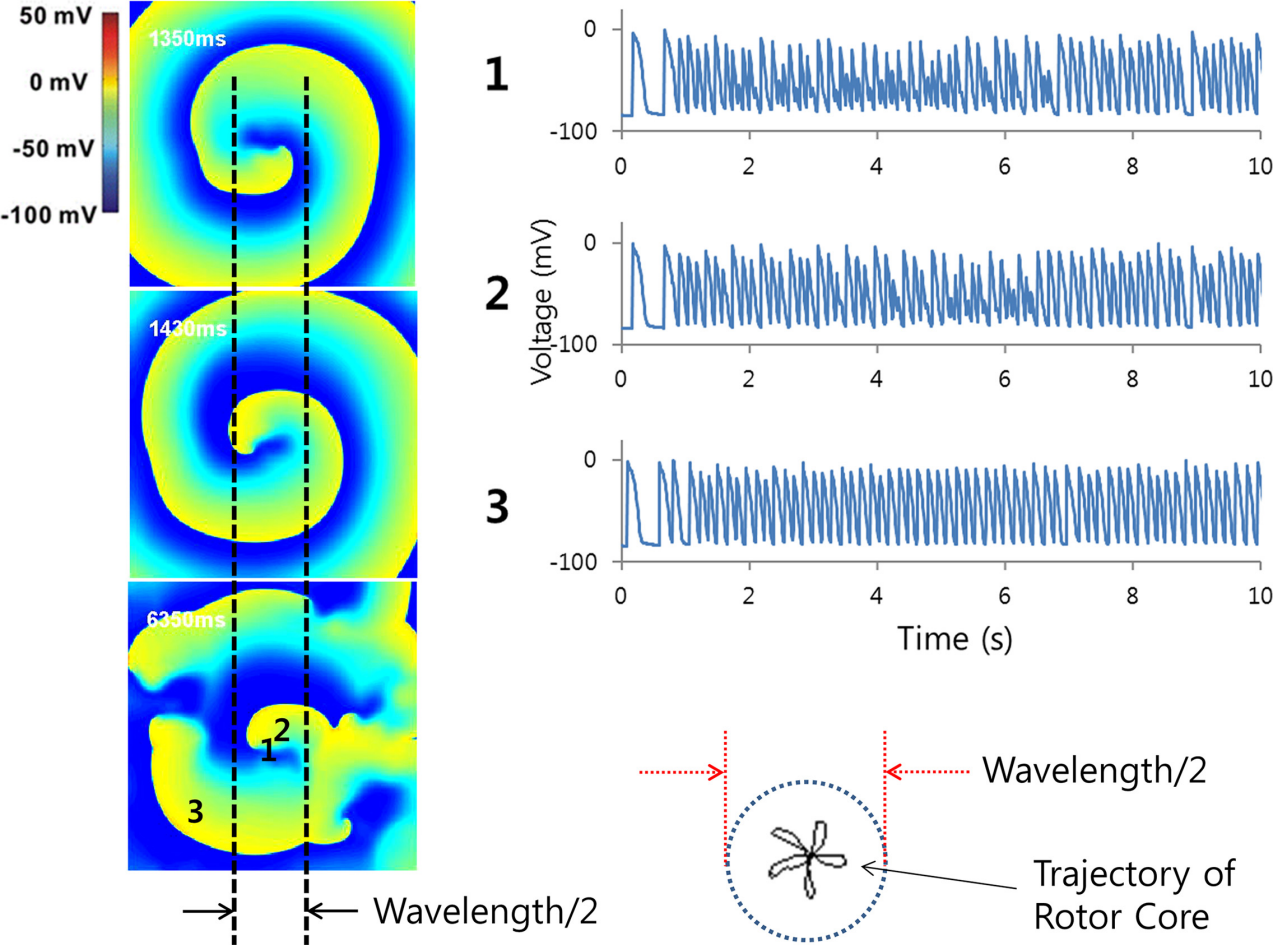
Spatial definition of mother rotor. The trajectory of a mother rotor tip remains within a circle of a diameter that is half the wavelength. Action potentials at three different locations (the center and periphery of the mother rotor tip trajectory and the periphery of the mother rotor) are also shown.

The mother rotor is defined as the rotor with its tip that remains within a circle of a diameter that is half the wavelength of the cardiac wave for longer than 5 s (Fig 1). The wavelength was determined for the linear wave generated by a line pacing with a 600-ms cycle length before reentry initiation. The rotor tip was defined as the PS point of the rotor. The rotor area was defined as the area bounded by a circle with a center and radius defined as follows. The center was determined as the mean coordinates of all PS points constituting the rotor. The radius was defined as the distance between the center and the PS point (constituting the rotor) located farthest from the center. Without a peripheral wave breakup, stable stationary reentries did not count as mother rotors.

### Virtual Ablations

For virtual ablations, the membrane potential of the ablated area was permanently set to the resting value to generate conduction block. Although the membrane potential should be set to zero and the inter-cellular coupling should be abolished in the ablated area, the potential was instead set to the resting value in this study. For DF- and ShEn-based ablations, the area (5%) with the highest values was ablated. For CFAE-based ablation, the area (5%) with the lowest CFAE-CL values was ablated. For the PS-based ablation, all the cells within 1 mm distance from a PS point were ablated considering the size of the tip of the ablation catheter. The PS points were ablated sequentially in time until the total ablation area was 5% of the whole computational domain.

### Three-Dimensional AF Modeling

A 3D simulation of AF was performed as previously described by Hwang et al. [20]. Briefly, a 3D electroanatomical map (NavX, St. Jude Medical Inc., Minnetonka, MN, USA) was generated using a circular pulmonary vein (PV) mapping catheter (Lasso, Biosense-Webster Inc., Diamond Bar, CA, USA). The NavX system-generated 3D geometry of the left atrium (LA) and PVs was merged with the corresponding 3D spiral computed tomography images. After smoothing the geometry, a 3D mesh was generated and refined on the geometry with a prism-type element using mesh generation software (HyperMesh, Altair, Troy, MI, USA). The LA appendage and myocardial sleeves were included in the mesh. Eq (1) was solved numerically on the 3D mesh using the finite element method to obtain the time-dependent membrane potential distribution. To calculate the ionic currents, the Courtemanche et al. [17] model of human atrial AP was used as in the simulation. An electrical stimulation was applied at the location of Bachmann’s bundle, and reentry was initiated by a ramp pacing in which a total of 40 pacings were applied with pacing intervals of 200, 190, 180, 170, and 160 ms.

## Results

### Correlations between the Mother Rotor and the DF, ShEn, or CFAE in 2D AF Models

**Fig 2:**
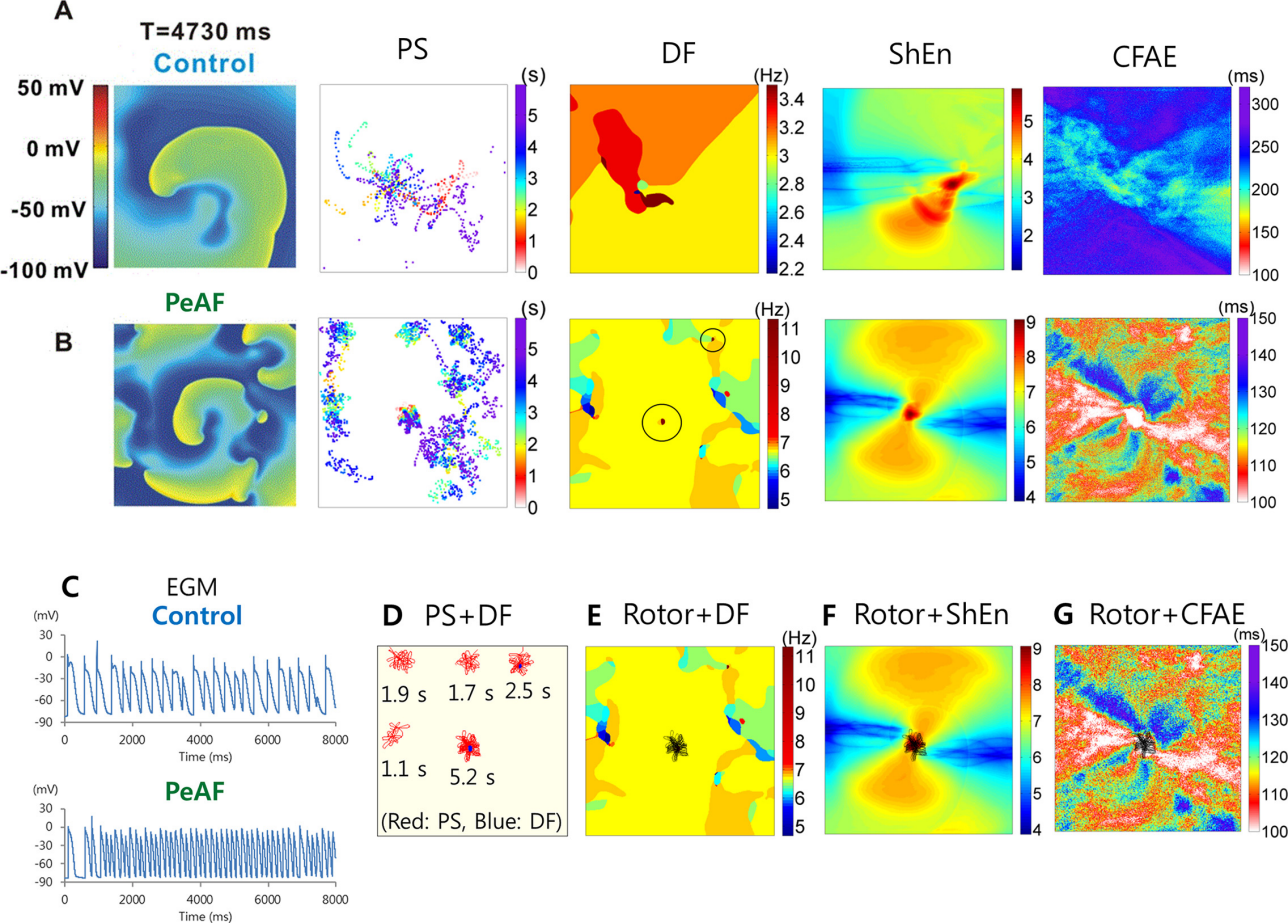
Cardiac wave dynamics around the mother rotor. Maps of voltage, PS, DF, ShEn, and CFAE under the control condition (A), at which ion current conductance values are the Courtemanche et al. [17] model values, and under AF condition (B), at which ion current conductance values are adjusted as shown in Table 1. In the DF map of the AF condition, the two highest DF areas are circled. Action potential curves are also shown for both control and AF conditions (C). D. DF areas (blue) are completely covered by the rotor tip trajectories (red). E. The trajectory of the mother rotor tip (black) is overlaid on the DF map. F. The trajectory of the mother rotor tip (black) is overlaid on the ShEn map. G. The trajectory of the mother rotor (black) tip is overlaid on the CFAE map.

A mother rotor, defined as that with its tip remaining within a circle of a diameter that is half the wavelength for longer than 5 s, was observed in the homogeneous 2D simulation (Fig 2). Wave breakups were induced at the wavefront of the mother rotor under AF condition, while under control conditions, the rotor did not generate actively breaking disorganized wavelets. Analyses of the PS points identified a single rotor that satisfied the rotor definition under both 2D control and AF conditions (Fig 2A and 2B).

Fig 2 also shows maps of the voltage, DF, ShEn, and CFAE as well as the AP curve at a spatial location (Fig 2C). Fig 2D shows the overlaying trajectories of the rotor tips on the areas of high DF. Of the two distinct high DF areas in Fig 2B, the area at the center (11.2 Hz) was completely included in the rotor area, while the smaller high DF area on the upper right side (11.3 Hz) remained in the area of stable reentry lasting longer than 2.5 s. The DFs of the areas corresponding to the other stable reentries were lower than the peak DF values on account of the shorter rotor maintenance times. Table 2 shows the overlap between the rotor area and the areas of DF, ShEn, and CFAE-CL. As the high DF areas were localized at the distinct two small areas (Fig 2B), we determined DF area based on DF>9Hz in the 2D model. For ShEn and CFAE-CL, we compared the upper 2.5% ShEn area and the lower 2.5% CFAE-CL area, as the mother rotor area was close to 2.5% of the whole tissue area. The rotor tip trajectory was found to overlap 71% of the high DF area (>9Hz, Fig 2E), 33.2% of the upper 2.5% of the ShEn area (Fig 2F), and 23.7% of the lower 2.5% of the CFAE-CL area (Fig 2G; p<0.001). Table 3 shows the percentages of overlapping areas among the areas of DF, ShEn, and CFAE-CL. The relationship between the high ShEn area and the low CFAE-CL area was most significant (p<0.001). AP morphology is highly disorganized at the highest DF area, which is located at the center of the rotor tip trajectory. AP morphology becomes more organized, and less frequency peaks are observed in the power spectra as the distance from the highest DF area increases (S1 Fig) [21, 22].

**Table 2:** Rotor area overlapped by the areas of DF, ShEn, and CFAE.

	DF area overlapped by Rotor area	ShEn area overlapped by Rotor area	CFAE-CL area overlapped by Rotor area	p-value
2D	71.0% (>9Hz)	33.2% (upper 2.5%)	23.7% (lower 2.5%)	< 0.001
3D	39.7% (>5.5Hz)	15.1% (upper 8.5%)	8.0% (upper 8.5%)	< 0.001

**Table 3:** Overlap of the areas of DF, ShEn, and CFAE (10%).

	DF area overlapped by ShEn area	ShEn area overlapped by CFAE area	CFAE area overlapped by DF area	p-value
2D	1.7%	15.3%	6.3%	< 0.001
3D	24.2%	23.0%	8.0%	< 0.001

### Correlations between the Mother Rotor and the DF, ShEn, or CFAE in 3D AF Models

**Fig 3:**
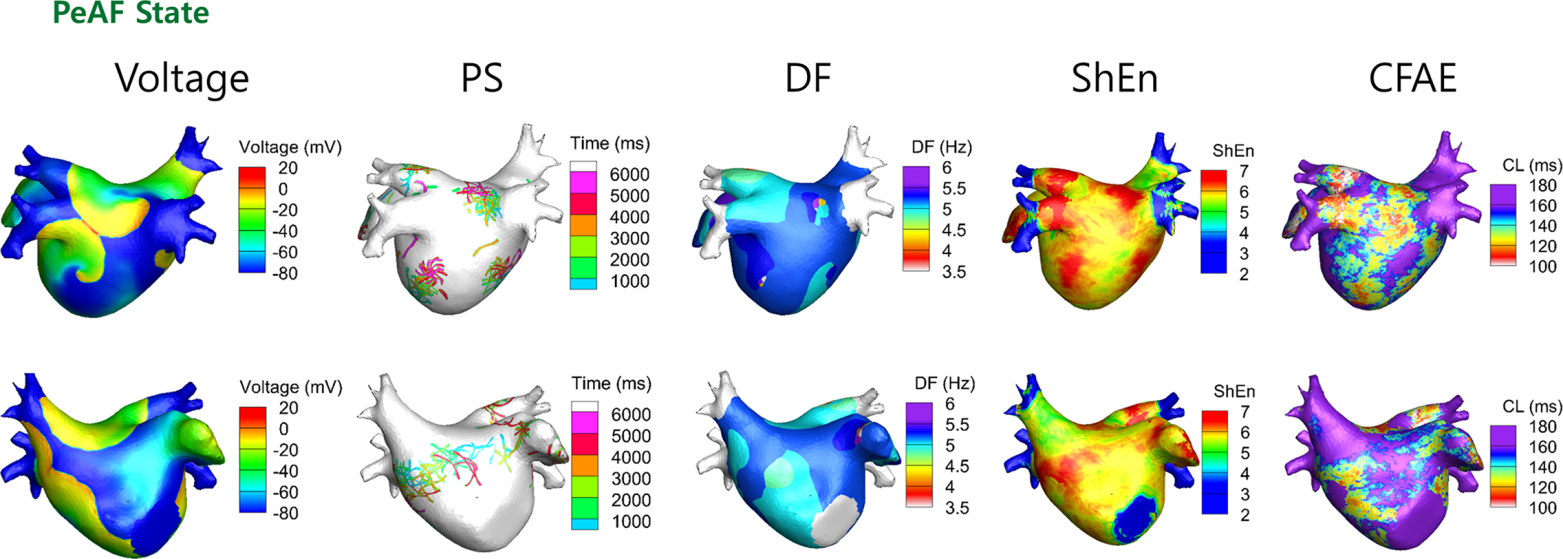
Three-dimensional maps of wave dynamics parameters. Maps of voltage, PS, DF, ShEn, and CFAE are shown for the case in which mother rotor was observed. The PS, DF, ShEn, and CFAE were mapped for 6 s.

Fig 3 shows PS, DF, ShEn, and CFAE maps during AF in 3D simulation. Three rotors were observed based on PS. The high DF area was determined by a cut-off DF value of 5.5 Hz based on the color coded 3D DF map. As the rotor area was 8.5% of the total surface area of the LA, we considered the upper 8.5% of the ShEn area and the lower 8.5% of the CFAE-CL area. The percentages of areas overlapping the rotor area were 39.7% for the high DF area, 27.5% for the ShEn area, and 9.9% for the CFAE-CL area (p<0.001; Table 2). The percentages of overlap were 24.2% between the high DF and high ShEn areas, and 23.0% between the high ShEn and low CFAE-CL areas. However, there was poor overlapping between the low CFAE-CL and high DF areas (8.0%, p<0.001; Table 3).

### DF Ablation Terminates AF

**Fig 4:**
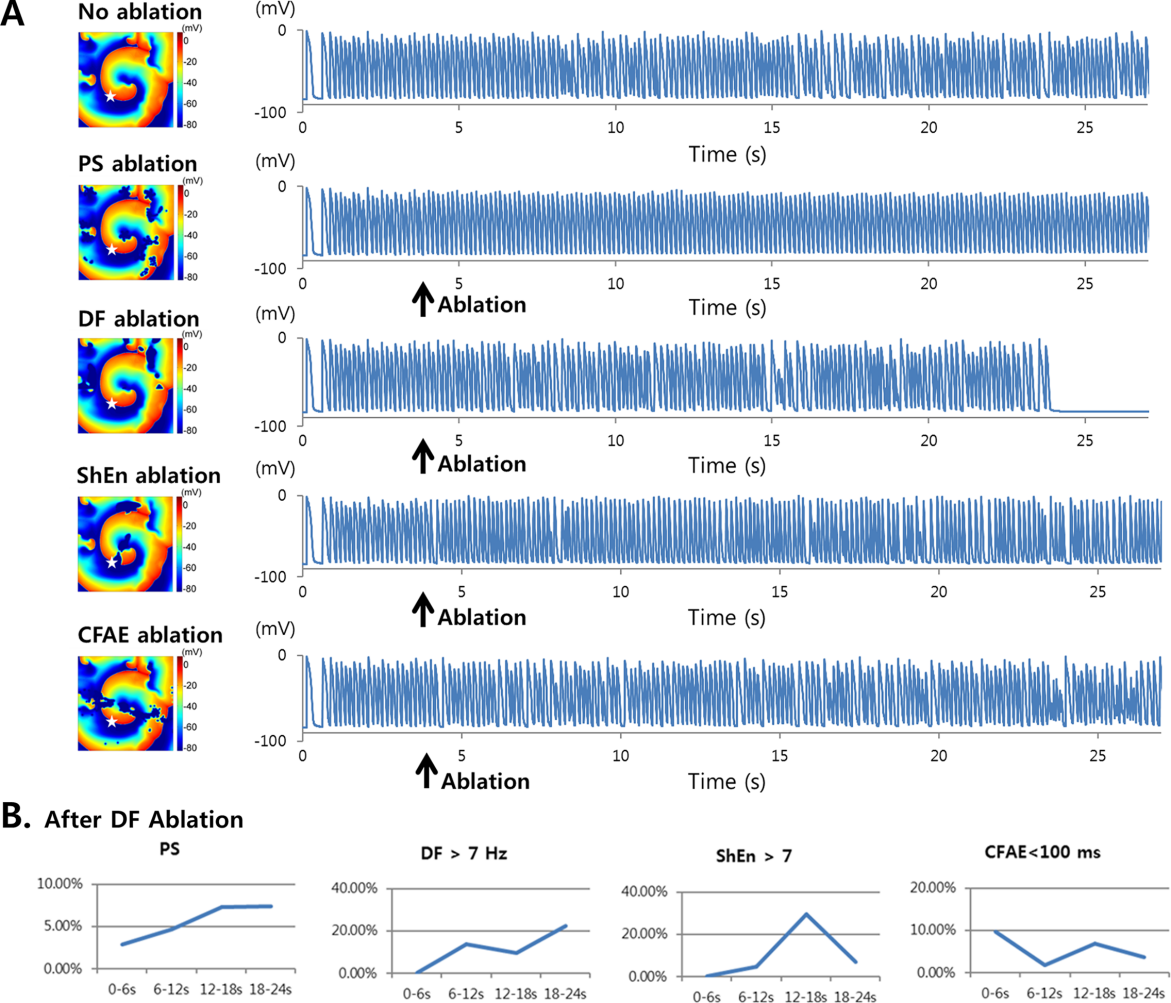
Wave dynamics change after virtual ablation. A. Action potential curves on the PS, DF, ShEn, and CFAE maps. Virtual ablation was performed at 4 s. Voltage maps show the ablated area. DF-based ablation terminated AF at 24 s. B. The changes in areas of PS, DF >7 Hz, ShEn >7, and CFAE <100 ms over time. White star indicates AP recording site.

In the 2D simulation, when PS-based ablation was performed for AF following the protocol described in the Methods section (Virtual Ablations), the rotor circled the ablated area and no longer induced wave breakups at the wavefront, thereby disqualifying the rotating wave as a mother rotor (Fig 4). PS-based ablation changed AF to the atrial tachycardia state. By contrast, DF-based ablation terminated AF at 20 s after ablation (Fig 4A, S1 Movie). ShEn- and CFAE-based ablations did not terminate AF and did not induce noticeable changes in wave dynamics. Fig 4B shows the changes in PS, DF, ShEn, and CFAE after DF-based ablation in 6-s intervals. The area where ShEn was greater than 7 increased transiently; however, this increase was followed by a decrease immediately before AF termination by DF-based ablation.

**Fig 5:**
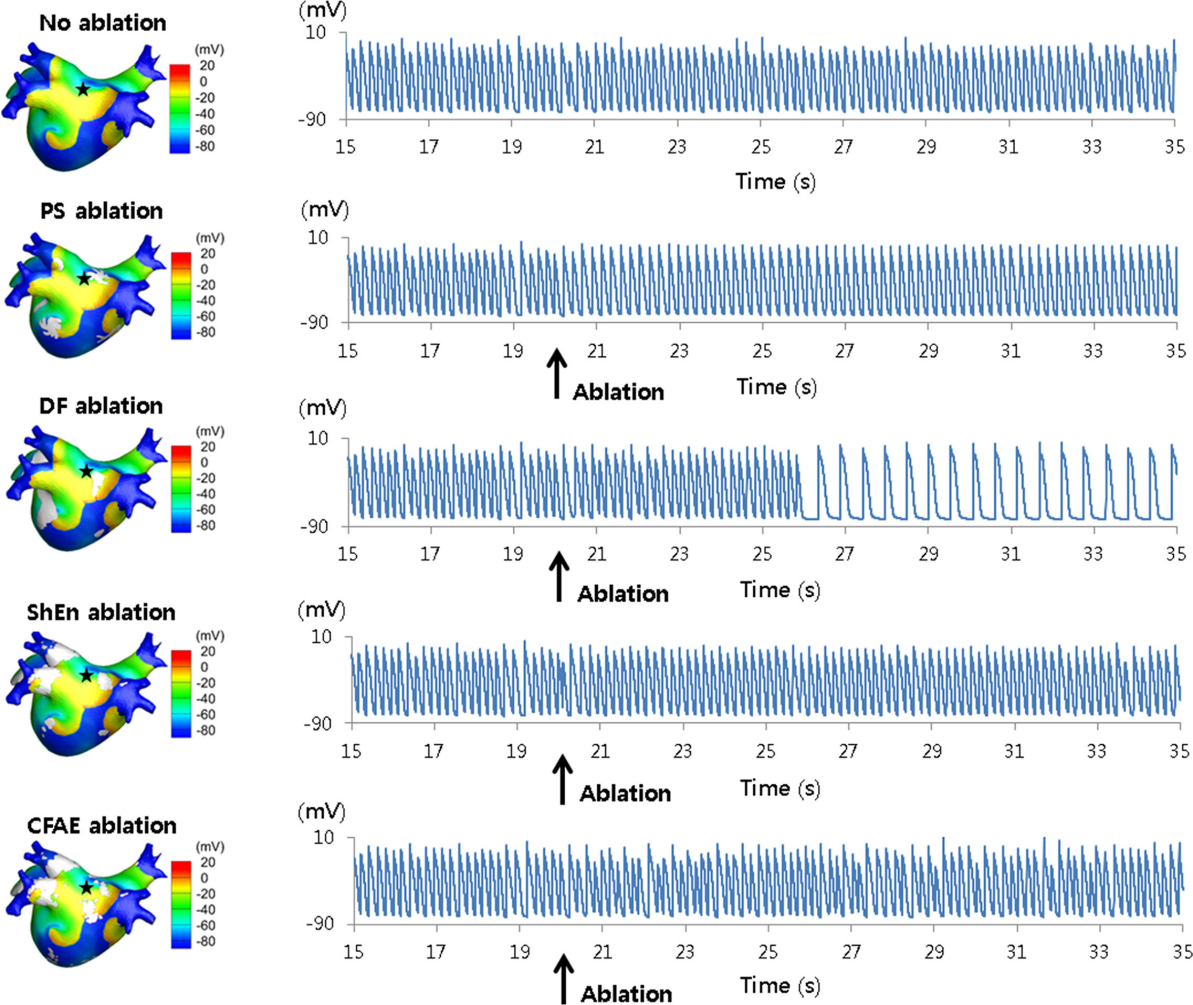
Action potential curves and 3D voltage maps for virtual ablations. PS-, DF-, ShEn-, and CFAE-based ablations were performed. The virtual ablations were performed at 20 s. DF-based ablation changed AF to AT. Black star indicates AP recording site.

The outcome of rotor ablation in the 3D simulation was consistent with that in the 2D simulation (Fig 5). PS-based ablation in the 3D model showed rotating waves around the ablated areas without wave breakups at the wavefronts, resulting in atrial tachycardia. DF-based ablation terminated AF approximately 7 s after ablation, leaving a slow atrial tachycardia with a 530 ms cycle length. As in the 2D simulations, the ShEn- and CFAE-based ablations did not alter wave dynamics. To test the reproducibility of the results of DF-based ablation, virtual ablation was performed on the models of 10 patients, seven of which exhibited conversion to regular AT (six) or termination of AF (one) (S2 Fig).

## Discussion

In this study, we defined the rotor both spatially (tip within a circle of a diameter that is half of the wavelength) and temporally (lasting longer than 5 s), and generated rotors *in-silico* in both 2D and 3D AF models. We examined PS, DF, ShEn, and CFAE near the rotor, and found that the high DF area was well-correlated with the rotor area. We performed virtual ablation based on PS, DF, ShEn, and CFAE in both the 2D and 3D models. Only DF-based ablation resulted in AF termination (2D model) or a change of AF to slow atrial tachycardia (3D model). In both the 2D and 3D models, PS-based ablation generated fast atrial tachycardia with multiple rotors circling the ablated area without wave breakups. Stable mother rotors do exist in both 2D and 3D human AF models. The locations of rotors were well represented by DF, and their virtual ablation altered wave dynamics and terminated AF.

### Rotor Presence as a Mechanism of AF

Mother rotors have been considered a mechanism of AF initiation and maintenance for the past few decades, though the concept was initially proposed a century ago [8–11]. Much experimental evidence and *in silico* validations have supported the role of mother rotors in AF [8–10]. Recently, Narayan et al. [23] observed rotors in AF patients, and found that ablation of the rotor is effective in terminating AF. Although more clinical studies are needed to confirm the existence of mother rotor and the effectiveness of rotor ablation in human AF with a higher resolution mapping, experimental and *in-silico* studies of AF rotor ablation are warranted as well. In this study, we examined the performance of various rotor ablation approaches that utilized different electrophysiological wave dynamics parameters to determine the ablation location and area. We analyzed multiple parameters using a virtual AP (PS and DF) or virtual bipolar electrogram (ShEn and CFAE) [13] under the same AF conditions. We also mapped and ablated the electrophysiological rotor. The *in-silico* observation in this study that ablation based on DF terminated AF suggests that mother rotors are very likely a mechanism of AF maintenance considering that the area of highest DF coincides with the mother rotor area. This antiarrhythmic effect of DF-based ablation is consistent with the results of several studies reported in the literature [11].

### PS Ablation vs. DF Ablation Targeting Rotors

Theoretically, AF can be terminated via limited ablation, targeting heterogeneities in tissue excitability, slow-conducting channels, and obstacles that are increasingly detectable [24, 25]. In the current study, there are several potential mechanisms for AF termination after DF ablation: First, the ablation lesion at the center of the mother rotor destabilizes the rotor, which eventually disappears (S3 Fig). Second, DF ablation removes reentries inside unexcitable ablated areas and reduces the number of PSs. Third, multiple-site DF area ablations terminated AF rather than generated stable reentries anchored to a single ablation area [26], as certain lesions were too small to act as an anchor while other lesions were located close to one another, which increased the instances of collisions among reentries. The PS-based ablation, which ablated the trajectories of rotor tips, altered the fibrillatory wave dynamics to tachycardia, supports the significance of the rotor as an ablation target and an AF maintenance mechanism. Although PS-based ablation did not eliminate reentry, the conversion of AF to anatomic reentry by focal ablation of a rotor is consistent with Spector’s study [27]. Spector observed that focal ablation of a rotor converts the functional block at its core into a structural block with the rotor still rotating around the block [27]. Although DF sites were proven to be associated with the locations of the mother rotor in VF [28], the applicability of this association to AF has been unclear. In this *in-silico* study, the mother rotor tip trajectory matched the highest DF site, and DF-based ablation terminated or converted AF to regular AT in 7 out of 10 patients suggesting that mother rotor may be the maintenance mechanism of AF. In our previous study on ventricular fibrillation wave dynamics, no correlation was found between DF and PS [13]. In the PS maps generated in the study (Fig 2 in [13]), a star-shaped PS trajectory, which is a characteristic of rotors, was rarely observed, but randomly meandering PSs dominated [13]. It can thus be speculated that the correlation observed in the present study between DF and PS was due to the rotors, which were dependent on ionic current states. The change in wave dynamics after virtual DF-based ablation shows a disorganized pattern of PS and ShEn increases and a decrease in CFAE-CL before AF termination preceded by an abrupt reduction of ShEn. The ablation of the mother rotor altered wave dynamics to a temporary disorganized rhythm, and termination followed when the driver was no longer present. This appears to support the focal source hypothesis of AF. However, the model used in this study was a homogeneous one reflecting pure physiological spiral wave dynamics, and DF-based ablation targeted not only the center of the mother rotor but also the upper 5% of the high DF sites. DF-based ablation limited to the mother rotor did not terminate AF in our models. The recent clinical study by Narayan et al. [23] showed somewhat contrary results to ours in that rotor-only ablation terminated AF in that study. More studies incorporating myocardial fiber orientation seem to be needed to clarify the association between DF-based ablation and rotor ablation.

### Clinical Implications

Narayan et al. [23] reported that a rotor was observed in human AF, and that AF catheter ablation targeting the rotor improved clinical outcomes. However, the spatial resolution was limited because AF mapping was performed using a 64-electrode basket catheter. Ganesan et al. [15] used ShEn to detect the rotor with a bipolar electrogram, which is typically used in the clinical environment. Haissaguerre et al. [29] reported that 75% of persistent AF was terminated by ablating noninvasively identified drivers, of which 80.5% were reentries and 19.5% were focal breakthroughs. Although there have been clinical reports of successful rotor-guided ablation in humans AF, the majority of patients had a prior history of AF ablation [23, 30] and there is a degree of controversy as to its effectiveness [31]. The recent RADAR-AF trial also failed to prove the superiority of DF-guided ablation compared to conventional ablation [32]. Sequential data point acquisition may raise concerns regarding DF stability, and it is difficult to use electrogram data (as it does not natively have the sinusoidal appearance of optically mapped data) [33] to localize DF in human AF [32, 34]. Therefore, conditions of clinical rotor- or DF-guided ablation are different from those of the simplified homogeneous model of the present study in which the entire LA is simultaneously mapped. More studies appear to be needed to confirm the proper locations for rotor ablation. Although the previous clinical study and our simulation study demonstrated the effectiveness of rotor ablation in AF termination, AF termination may not improve the long-term clinical outcome of AF ablation as reported by other studies [29]. Since Konings et al. [35] introduced CFAE mapping, Nademanee et al. [16] reported that CFAE ablation increased AF termination. However, a recent multicenter randomized trial (STAR-AF2) and meta-analysis [36] showed that CFAE-based ablation did not improve clinical outcomes significantly. Although VF can be terminated using catheter ablation targeting the mother rotor indicated by the highest DF [4, 5], the outcomes of rotor ablation have not been consistent under AF conditions despite the rotor being an evident mechanism. The reasons for this seem to be 1) there has been no clear and consensus definition of a rotor, 2) the structure of the atrium is more complex than that of the ventricle, and 3) the change in temporal wave dynamics is excessive due to electrical and structural remodeling. A fast and reproducible method of clinically mapping the AF rotor would improve AF catheter ablation outcomes. Virtual ablation using simulation modeling can be useful in this regard [20], and would be valuable for predicting the risk of arrhythmia [37].

### Limitations

This study has some limitations. The 3D model used in this study is a model of a single patient. Another limitation is that the 3D model used in this study is a structurally homogeneous left atrial model excluding the effects of histological changes; however, such a model represents a pure electrophysiological rotor. Bi-atrial application, thickness variation, and structural characteristics such as fiber orientation could affect wave propagation. However, wave propagation in the monolayer model was reported to be similar to that in a bilayer model except for the area of abrupt change of fiber orientation [38], not altering the main findings of the present study. Additionally, the ionic current properties are spatially uniform in this model. Experimental and simulation studies, however, have shown that heterogeneity plays a major role in determining rotor dynamics resulting in drifting and peripheral wave breakups [39]. Pandit et al. [14] observed an inverse relationship between rotor DF and the meandering area of the rotor. Although CFAE signals are dependent on electrode configuration, we used a single type of electrode to generate virtual CFAE, as CFAE has been utilized in clinical catheter ablation without considering electrode configuration. The electrograms were obtained by averaging the action potentials of the cells covered by the electrodes. Although similar approximation was used by Pertsov et al. [40], more standard methods such as the one by Weinberg et al. [41] and Ganesan et al. [42] would provide more realistic electrograms. This may explain the difference in the effectiveness of DF ablation in clinical studies [32, 34] and preclinical studies using optically mapped cellular action potential [43].

## Conclusion

Mother rotors, which are spatiotemporally stable and induce wave breakups, were found to emerge in a human *in-silico* AF model depending on electrophysiological conditions, and the location of the mother rotor tip trajectory was well represented by DF. Virtual ablation based on DF altered wave dynamics and terminated AF.

## Supporting Information

S1 Fig AP curves and power spectra on DF map. AP curves and power spectra are shown at the highest DF site (1), at a location near the highest DF site (2), and at a location far from the highest DF site (3).
(DOCX)

S2 Fig Virtual DF-based ablation performed on the models of 10 patients. DF maps (both anterior and posterior sides) are shown on the left panel. Action potential curves for each of the 10 patients are shown on the right panel. Black star indicates action potential recording site.
(DOCX)

S3 Fig PS maps after DF ablation. The small ablation lesion at the center destabilizes the mother rotor. The ablation removed the PSs that were previously located in the areas of ablation lesions. The ablation decreased the total number of PSs more so than no ablation. No anchored reentry around any of the ablation lesions was observed.
(DOCX)

S1 Movie Termination of AF by DF ablation. DF ablation was applied at 4 s. AF was terminated at approximately 24 s.
(AVI)
