# Supplementary material for: Electrophysiological Rotor Ablation in In-Silico Modeling of Atrial Fibrillation: Comparisons with Dominant Frequency, Shannon Entropy, and Phase Singularity
Source: PLoS One. 2016 Feb 24;11(2):e0149695. doi: 10.1371/journal.pone.0149695 (PMC4766081; doi:10.1371/journal.pone.0149695)
Supplement: S1 Fig — AP curves and power spectra are shown at the highest DF site (1), at a location near the highest DF site (2), and at a location far from the highest DF site (3). (DOCX) [file pone.0149695.s001.docx]

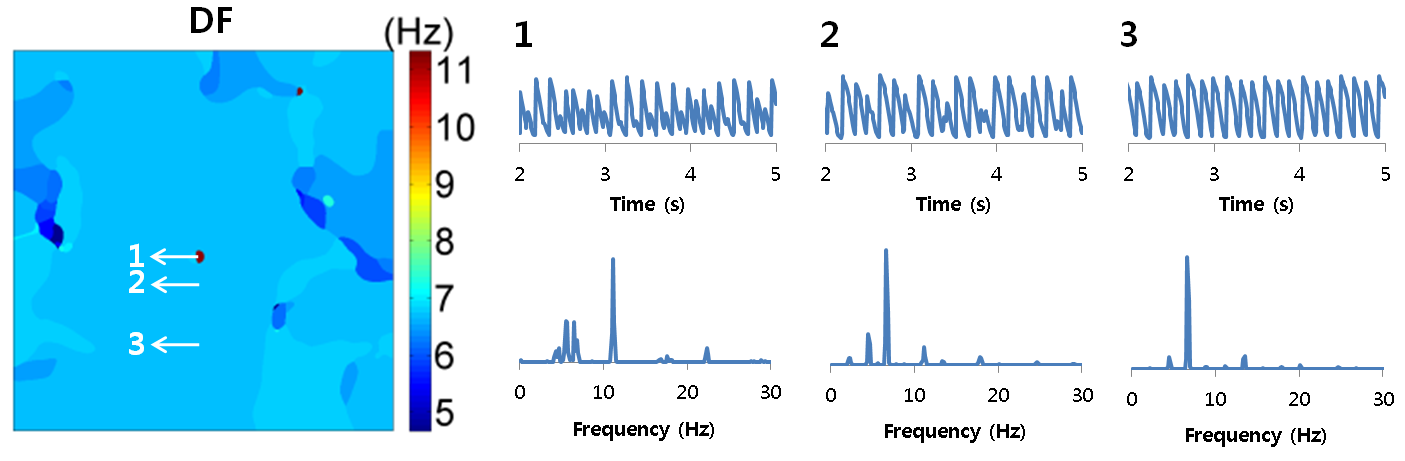


**S1 Fig**. AP and power spectra at the highest DF site (1), at a location near the highest DF site (2), and at a location far from the highest DF site (3).
