# Supplementary material for: Electrophysiological Rotor Ablation in In-Silico Modeling of Atrial Fibrillation: Comparisons with Dominant Frequency, Shannon Entropy, and Phase Singularity
Source: PLoS One. 2016 Feb 24;11(2):e0149695. doi: 10.1371/journal.pone.0149695 (PMC4766081; doi:10.1371/journal.pone.0149695)
Supplement: S2 Fig — DF maps (both anterior and posterior sides) are shown on the left panel. Action potential curves for each of the 10 patients are shown on the right panel. Black star indicates action potential recording site. (DOCX) [file pone.0149695.s002.docx]

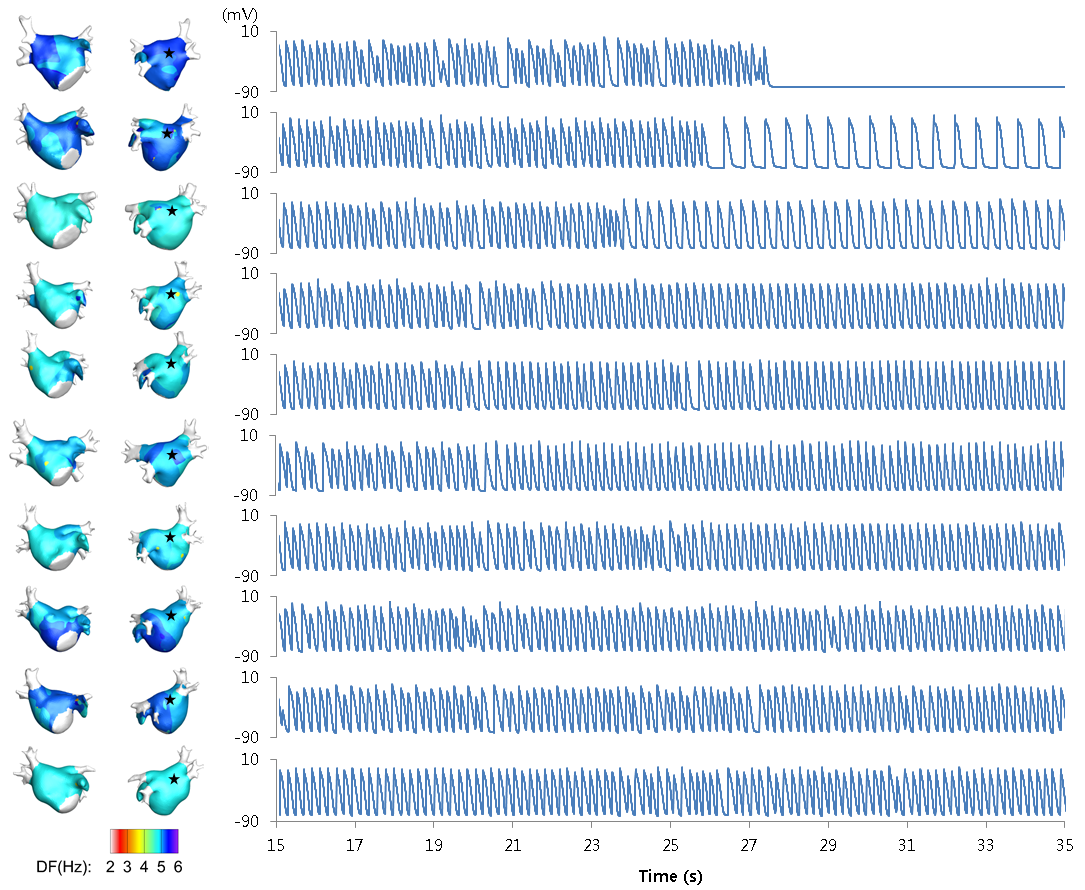


**S2 Fig**. Virtual DF-based ablation performed on the models of 10 patients. DF maps (both anterior and posterior sides) are shown on the left panel. Action potential curves for each of the 10 patients are shown on the right panel. Black star indicates action potential recording site.
