# Supplementary material for: Electrophysiological Rotor Ablation in In-Silico Modeling of Atrial Fibrillation: Comparisons with Dominant Frequency, Shannon Entropy, and Phase Singularity
Source: PLoS One. 2016 Feb 24;11(2):e0149695. doi: 10.1371/journal.pone.0149695 (PMC4766081; doi:10.1371/journal.pone.0149695)
Supplement: S3 Fig — The small ablation lesion at the center destabilizes the mother rotor. The ablation removed the PSs that were previously located in the areas of ablation lesions. The ablation decreased the total number of PSs more so than no ablation. No anchored reentry around any of the ablation lesions was observed. (DOCX) [file pone.0149695.s003.docx]

**
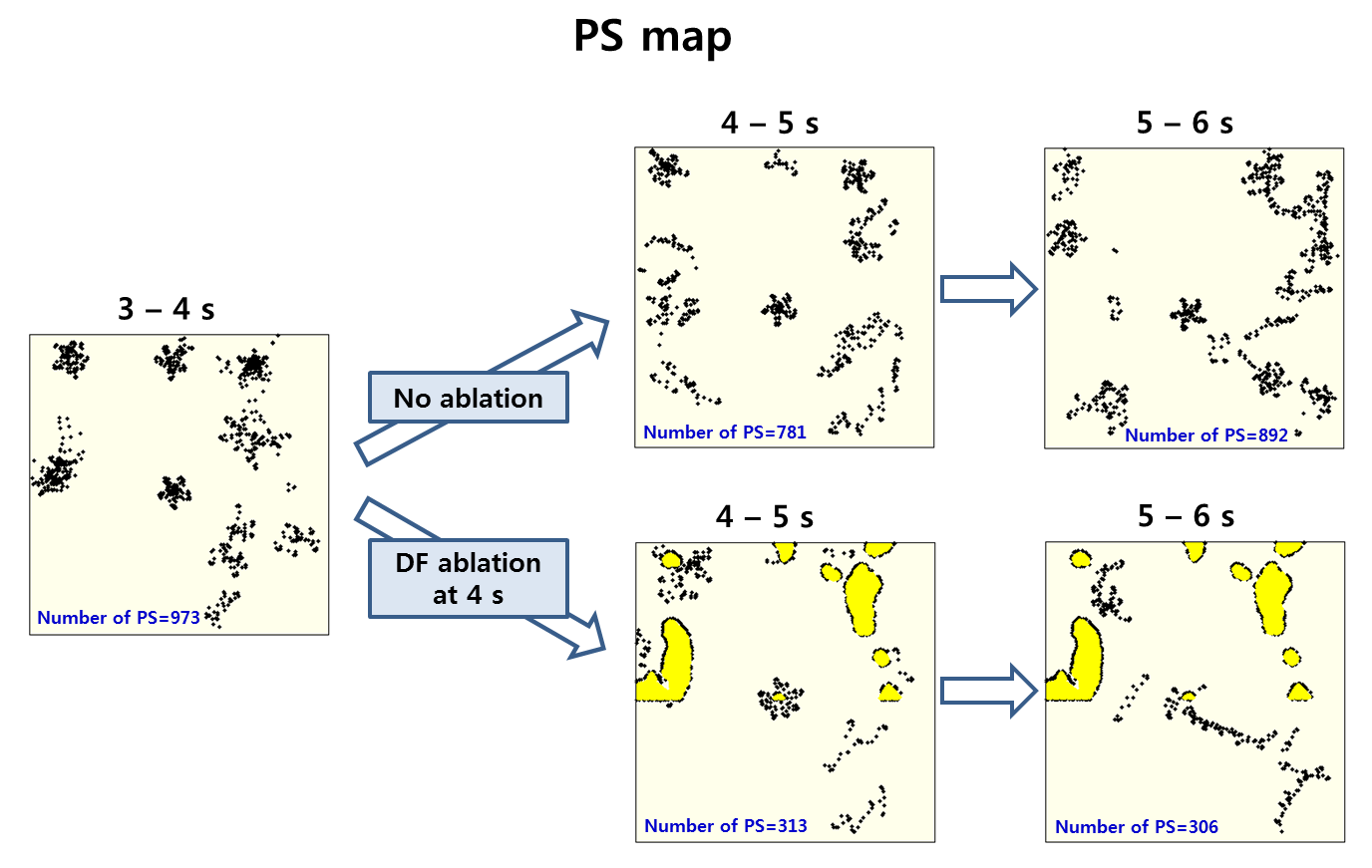
**

**S3 Fig**. Virtual DF PS maps after DF ablation. The small ablation lesion at the center destabilizes the mother rotor. The ablation removed the PSs that were previously located in the areas of ablation lesions. The ablation decreased the total number of PSs more so than no ablation. No anchored reentry around any of the ablation lesions was observed.
